# The Role of Infection and Inflammation in Stillbirths: Parallels with SIDS?

**DOI:** 10.3389/fimmu.2015.00248

**Published:** 2015-06-09

**Authors:** Caroline Blackwell

**Affiliations:** ^1^School of Biomedical Sciences, Faculty of Health and Medicine, University of Newcastle, Newcastle, NSW, Australia; ^2^Information Based Medicine, Hunter Medical Research Institute, New Lambton, NSW, Australia

**Keywords:** stillbirth, sudden infant death syndrome, inflammation, infection, cigarette smoke, ethnicity, obesity

## Abstract

It has been suggested that stillbirths are part of the spectrum of infant deaths that includes sudden infant death syndrome (SIDS). This paper examines the hypothesis that risk factors associated with stillbirths might contribute to dysregulation of inflammatory responses to infections that could trigger the physiological responses leading to fetal loss. These include genetic factors (ethnic group, sex), environmental (infection, cigarette smoke, obesity), and developmental (testosterone levels) factors. Interactions between the genetic, environmental, and developmental risk factors are also considered, e.g., the excess of male stillborn infants in relation to the effects of testosterone levels during development on pro-inflammatory responses. In contrast to SIDS, inflammatory responses of both mother and fetus need to be considered. Approaches for examining the hypothesis are proposed.

## Infection and Inflammation in Infant Deaths

There have been suggestions that stillbirths are part of the spectrum of infant deaths associated with sudden infant death syndrome (SIDS) based on epidemiological parallels (1). Some of the reported parallels included: ethnic background; maternal smoking; small for gestational age infants; evidence of infection/inflammation in mother and/or fetus. Infections have been implicated in the etiology of stillbirths in both developing and industrialized countries (2–4). As with SIDS and sudden unexpected deaths in infancy (SUDI), no single organism has been implicated (5–7). The common thread to be considered in this review is the inflammatory responses to infection and how the risk factors identified in epidemiological studies might affect these responses in both mother and infant. Based on our previous work on SIDS, our hypothesis is that genetic and environmental risk factors that result in dysregulation of inflammatory responses by mother and/or infant to infection could contribute to events leading to some unexplained stillbirths. Table 1 lists the major risk factors for SIDS and for stillbirths and cites the supporting literature. In the following sections, the effects of genetic and environmental factors on inflammatory responses will be assessed.

**Table 1:** **Comparison of risk factors for SIDS/SUDI and stillbirths**.

SIDS/SUDI	Reference	Stillbirths	Reference
Ethnic group	(8–10)	Ethnic group	(11–13)
Male gender	(14, 15)	Male gender	(16, 17)
Cigarette smoke	(18)	Cigarette smoke	(12)
Infection	(5, 19)	Infection	(3, 4)
Prematurity	(20)	Prematurity	(12)
Small baby	(20)	Small baby	(12)
Overweight/maternal obesity	(21, 22)	Overweight/maternal obesity	(23, 24)

### Infection in stillbirths

The incidence of stillbirths ranges from as few as 3/1000 births in developed countries to as many as 45/1000 in developing countries (25) where infection is more common.

Early studies implicated inflammation associated with infectious agents (26, 27). There are usually no overt signs of infection prior to fetal loss including: maternal fever or chills; abdominal discomfort; or fetal tachycardia. Ascending bacterial infection (before and after membrane rupture) was identified as the most common infectious cause of stillbirth. Infection can also occur from hematogenous spread (4). The most common organisms involved were *Escherichia coli*, group B *Streptococcus pyogenes*, and *Ureaplasma urealyticum*. The two most common viral infections associated with stillbirths were parvovirus and Coxsackie virus (3, 26). A more recent study identified cytomegalovirus (CMV) in 15% of stillbirths (28). Serological studies have implicated *Chlamydia trachomatis* in a Scandinavian study of stillbirths (29). Although infection is considered a common cause of stillbirth, it is often hard to attribute this causally for a number of reasons. Several groups have studied the use of polymerase chain reaction (PCR) to identify specific viral and bacterial DNA and RNA and have found it to be more sensitive than routine microbiological methods in detecting evidence of infection in stillborn babies (28, 30).

Both invasive and toxigenic bacteria need to be considered as bacterial exotoxins or their cellular components can act as superantigens eliciting strong pro-inflammatory responses. A comprehensive review of the literature relating to stillbirth/intrauterine fetal death (IUFD) and infection suggested that between 10 and 25% of all cases of IUFD in developed countries were associated with infection (4). We identified pyrogenic toxins of *Staphylococcus aureus* in serum or tissues of over 50% of SIDS infants from five different countries (31) and toxins of enteric organisms have also been implicated (32); however, there has been no systematic assessment of material from stillbirths for presence of bacterial toxins.

The mechanisms proposed for the role of infection in stillbirths include (1) maternal illness resulting in fever, respiratory distress, or systemic responses to the infection; (2) infection of the placenta resulting in reduced fetoplacental blood flow; (3) direct infection of the fetus; (4) induction of pre-term labor (4). Inflammation might contribute to all of these, and the inflammatory response of the fetus also needs to be considered.

## Inflammation and Infant Deaths

The inflammatory response is the major protective mechanism evolved to deal with pathogenic micro-organisms. The pro-inflammatory cytokines are involved in clearing the microorganism. The anti-inflammatory cytokines are involved in damping down the pro-inflammatory responses to prevent collateral damage of a too abundant response to infection; however, an innate tendency to enhanced anti-inflammatory signaling is thought to increase the risk of death through infection. Successful reproduction necessitates adequate immune tolerance to allow pregnancy to proceed without rejecting the fetus, half of whose antigens are from the father. Pro-inflammatory responses have been associated with increased resistance to infection and anti-inflammatory responses with increased fertility (33). Genetic and environmental factors that disturb the balance between pro- and anti-inflammatory cytokines might result in fetal damage. There is evidence that some ethnic groups at increased risk of infant deaths due to infection, SIDS or stillbirths (12, 23, 34). These include Indigenous communities in Canada and Australia and African-Americans (13, 23, 34). In these populations at higher risk of stillbirths, there is a general genetic predisposition to strong pro-inflammatory responses (35–40).

Women with low capacity to respond to vaginal infection through the production of pro-inflammatory cytokines, interleukin (IL)-1β, IL-6, and IL-8 might have a more permissive environment for pathogens to flourish and be at risk of ascending uterine infection and chorioamnionitis (41). Enhanced pro-inflammatory responses to vaginal infection or periodontal disease (42) are suggested to be detrimental to pregnancy and elevated levels of IL-6 have been found to be a predictor of pre-term labor (43, 44).

As with SIDS, histopathological changes in the placenta or fetus are not always consistent (45), and the presence of organisms does not always imply causation, although it is more likely if micro-organisms are found in fetal tissue compared with placenta or fetal membranes. Examination of placentas from live and stillborn infants found evidence of inflammation in 30.4% of stillbirths compared with 12% of controls. Inflammation was more common in placentas from early stillborn deliveries and also in early live births (46). Chorioamnionitis without fetal inflammatory responses was associated with stillbirth in early pre-term pregnancies (47).

**Figure 1:**
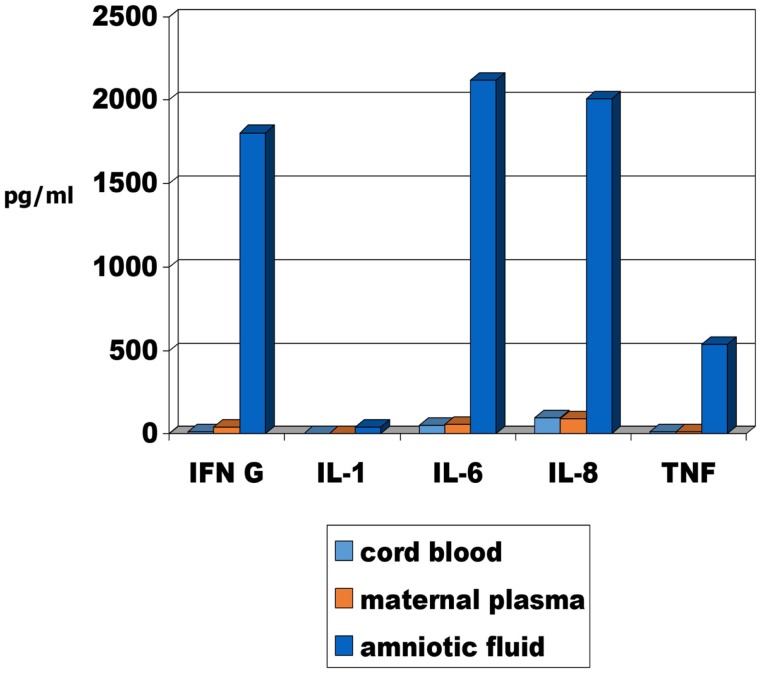
**Median pro-inflammatory cytokine levels in maternal plasma, cord blood and amniotic fluid of 24 mothers and infants obtained from elective Caeserean delivery [adapted from Burns et al. (49)]**.

It has been recommended that there is a need to assess the molecular evidence for inflammation in these deaths (48). In the case of SIDS, factors affecting the inflammatory responses of the infant need to be considered; for stillbirths, factors affecting the inflammatory responses of both mother and infant need to be considered. The methodology is available and preliminary studies on levels of cytokines in matched samples of maternal plasma, cord blood, and amniotic fluid from late pregnancy are reported in this issue (49). The levels of pro-inflammatory cytokines are significantly higher in the amniotic fluid compared with the levels in cord blood or maternal plasma (Figure 1).

## Assessment of Inflammatory Responses in Relation to Risk Factors

### Ethnic group

The incidences of infant mortality due to infection, SIDS and stillbirths are higher among families of Indigenous groups (e.g., Aboriginal Australians and Native Americans) compared to families of European origins in the same countries (Table 2).

**Table 2:** **Incidence of stillbirths among different ethnic groups**.

Country	No/1000 births
United kingdom (12)
European	3.2
Non-UK	4.0
African	7.4
Afro-Caribbean	6.7
Bangladeshi	4.2
Indian	
UK	3.9
Non-UK	6.4
Pakistani	
UK	4.1
Non-UK	6.9
Australia (11)
Non-Indigenous	5.9
Indigenous	9.1
United states (13)
Hispanic	5.44
Non-Hispanic white	4.75
Non-Hispanic black	11.13
Native American/Alaska Native	6.17

Ethnicity was a significant risk for stillbirth in the United Kingdom; those groups at increased risk included African, Afro-Caribbean, Indian, and Pakistani mothers. In other countries, Indigenous mothers have an increased risk for stillbirth. While these disparities have been ascribed primarily to socio-economic disadvantage, there is emerging evidence that genetic background (40) and interactions between environmental factors such as cigarette smoke might contribute to susceptibility to, and severity of, inflammatory responses to infections (37, 50).

In relation to potential underlying factors affecting inflammatory responses and their role in stillbirths, it is important to note that cytokine gene polymorphisms associated with high-level responses of pro-inflammatory cytokines or low-level responses of anti-inflammatory cytokines such as IL-10 are predominant among some Indigenous groups, South Asians, and American Black populations (36, 40, 51). There are experimental and epidemiological studies indicating that genetic polymorphisms in the inflammatory response might contribute to poor pregnancy outcome; however, the results are inconsistent. Pre-term labor enhances the risk of stillbirth (4). Important risk factors include intrauterine infection/inflammation and social factors (stress, smoking, heavy work). The final common pathway appears to be activation of the inflammatory cascade. Bacterial infection and/or inflammation of the choriodecidual interface induces pro-inflammatory cytokine responses leading to neutrophil activation, synthesis, and release of prostaglandins causing uterine contractions and metalloproteinases weakening fetal membranes (52). Polymorphisms associated with increased production of pro-inflammatory and/or decreased production of anti-inflammatory cytokines have been implicated in pre-term birth. Those enhancing the magnitude or duration of the responses were associated with risk of pre-term birth (53, 54). *In vitro* studies with leukocytes from women with recurrent pregnancy loss found significantly higher levels of interferon-γ (IFN-γ) and a trend toward increased TNF-α production compared with women with no history of pregnancy loss. In relation to IL-6 and TGF-β, no significant differences were detected between the groups (55).

## Modifiable Risk Factors and Inflammation

In relation to our previous studies on interactions between genetic and environmental factors on inflammatory responses, it is recommended that assessments of the genetic predisposition to inflammation need to control for environmental risk factors that alter cytokine responses to infection or toxins (37, 39). The major environmental factors to be considered in this review are co-infections, smoking, and obesity.

## Infections

Virus pandemics have been associated with increased risk of pre-term labor and fetal loss (56, 57). There are a number of models that indicate that virus infections can potentiate the effects of bacterial toxins implicated in SIDS (58–60). There is also a mouse model that found that while an asymptomatic infection with the murine gamma herpes virus 68 did not disrupt pregnancy outcome, the infection could upregulate the pro-inflammatory responses to small quantities of endotoxin in both placenta and decidua, resulting in pre-term labor and fetal loss. Similar responses were observed for human primary trophoblast and trophoblast cell lines infected with this virus prior to exposure to endotoxin (61). The enhancement of pro-inflammatory responses to endotoxin was attributed to priming by IFN-γ and TNF-α responses to the virus infection. Additional evidence for the role of IFN-γ was provided by *in vitro* studies with human monocytic cells and the THP-1 cell line (50, 62) (Moscovis et al., this issue).

Chronic infections such as *Helicobacter pylori*, *Chlamydia pneumoniae*, and CMV can also significantly increase pro-inflammatory markers (63). *H. pylori* infection is significantly higher among mothers with small for gestational age infants (64). In a population in India, periodontal disease was associated with increased levels of C-reactive protein (CRP) and also with pre-term birth (65).

## Cigarette Smoke

Both active smoking and passive exposure to cigarette smoke have been reported to enhance risk of stillbirth (12). Cigarette smoke can influence infection and inflammation is several ways: (1) enhanced susceptibility to respiratory virus infection and subsequent enhanced colonization by potential bacterial pathogens; (2) increase in the numbers and species of respiratory bacteria due to enhanced “stickiness” of epithelial cells coated with smoke components (66); (3) enhanced pro-inflammatory responses to bacterial antigens (50); (4) reduction in anti-inflammatory IL-10 responses (37).

IL-10 appears to protect the fetus against pathogens. IL-10 knockout mice are at greater risk of some pregnancy pathologies that occur in response to infection. Low doses of endotoxin given to IL-10 knockout mice can cause fetal resorption in early pregnancy (67) and pre-term labor in late pregnancy (68). No effect on pregnancy was observed when wild-type mice were given the same dose. IL-10 acts through inhibition of inflammatory cytokines including TNFα, IFN-γ, and IL-6 (67, 69).

The *IL10*-1082A alleles have been associated with reduced production of IL-10. One SNP (G-1082A) in the promoter sequence of the *IL10* gene associated with under-expression of plasma IL-10 levels (70, 71) was present in a significantly greater proportion of ethnic groups at increased risk of stillbirths: Black Americans (45%) (36), Bangladeshis (84%), and Aboriginal Australians (83%) compared with Caucasian populations (31%) (40). Smokers had significantly lower baseline levels of IL-10 and lower responses to endotoxin than non-smokers (37). When assessed by genotype, the differences between smokers and non-smokers were significant for individuals with the heterozygous variant (GA) and the variant (AA). These data suggest interactions between cigarette smoke and genetic factors that result in reduced control of pro-inflammatory responses by IL-10.

## Obesity

One of the latest meta-analyses of risk factors for stillbirths indicated that maternal overweight/obesity [body mass index (BMI) >25 kg/m^2^] was the highest modifiable risk factor with a population attributable risk (PAR) of 8–18% contributing to >8000 stillbirths across all high-income countries. Maternal smoking had a PAR of 4–7% contributing to more than 2800 stillbirths across all high-income countries (23). The physiological mechanisms contributing to stillbirths are not well defined; however, obesity increases the risk of gestational diabetes and hypertension. There is evidence to suggest inflammation is also involved.

Adipose tissue from lean individuals preferentially secretes anti-inflammatory adipokines such as adiponectin, transforming growth factor beta (TGFβ), IL-10, IL-4, IL-13, IL-1 receptor antagonist (IL-1Ra), and apelin. By contrast, obese adipose tissue mainly releases pro-inflammatory cytokines among which are TNF-α, IL-6, leptin, visfatin, resistin, angiotensin II, and plasminogen activator inhibitor 1 (72). In studies of obesity among Indigenous groups in which the genotype associated with higher levels of IL-6 responses is predominant, levels of this cytokine were associated with higher BMI (73, 74). Using CRP as a marker for inflammation, there is a positive correlation between BMI and CRP among adults (75). In our current studies, BMI correlated with CRP levels among Indigenous Australian women during pregnancy (Pringle, this issue).

## Fetal Growth Restriction

Fetal growth restriction had the largest PAR for stillbirths in a major study of still birth risk factors in the United Kingdom (12). Down regulation of IL-10 in the placenta has been associated with IUGR in studies of a Caucasian (Swedish) (76) and an Asian (Pakistani) (77) population. Elevated CRP (≥25 mg L^−1^) was associated with lower estimated fetal weight in the third trimester and lower weight at birth and an increased risk of a small for gestational age infant (78). In a mouse model, IL-10-reduced endotoxin-induced growth retardation and fetal deaths (79); and we have found a significant correlation (*r* = 0.91) between levels of maternal and cord blood IL-10 among matched samples from elective Caesarian deliveries (49).

Both human recombinant IL-10 and the CMV IL-10 analog down regulate matrix metalloprotein 9 (MMP 9) involved in implantation. Reduced MMP 9 activity in early placenta formation has been suggested to affect cytotrophoblast remodeling of the uterine vasculature and restrict fetal growth (80). There have been no prospective studies on presence of the levels of IL-10 or the presence of CMV IL-10 analog in human pregnancy outcome. It needs to be determined if these might be associated with low-birth weight or small for gestational age infants if there is a parallel with the mouse models. The report that 15% of stillbirths in one series had evidence of CMV infection warrants further studies into the role of these infections in relation to infection and inflammation on the outcome of pregnancy, fetal survival, and health (28).

There is evidence from animal models that elevated testosterone during pregnancy results in intrauterine growth retardation (81). Among women with polycystic ovary (PCO) syndrome, maternal androgens are increased during pregnancy. At 10–16 weeks, the PCO group had higher levels of testosterone and the differences were significant at 22–28 weeks (82). In the PCO mothers, there was a higher proportion of small for gestational age infants (12.8%) compared with the control group (2.8%); and the SGA infants of the mothers with PCO were significantly smaller (83). Higher levels of testosterone during pregnancy at 17 and 33 weeks were associated with lower birth weight and length of the infant. The levels ranged from 0.5 to 7.2 nMol L^−1^ at 13 weeks and from 0.9 to 14.5 nMol L^−1^ at 33 weeks (84). If inflammatory responses are contributing to growth restrictions, the effects of testosterone need to be considered in the context of inflammation (62).

## The Male Excess in Stillbirths

**Figure 2:**
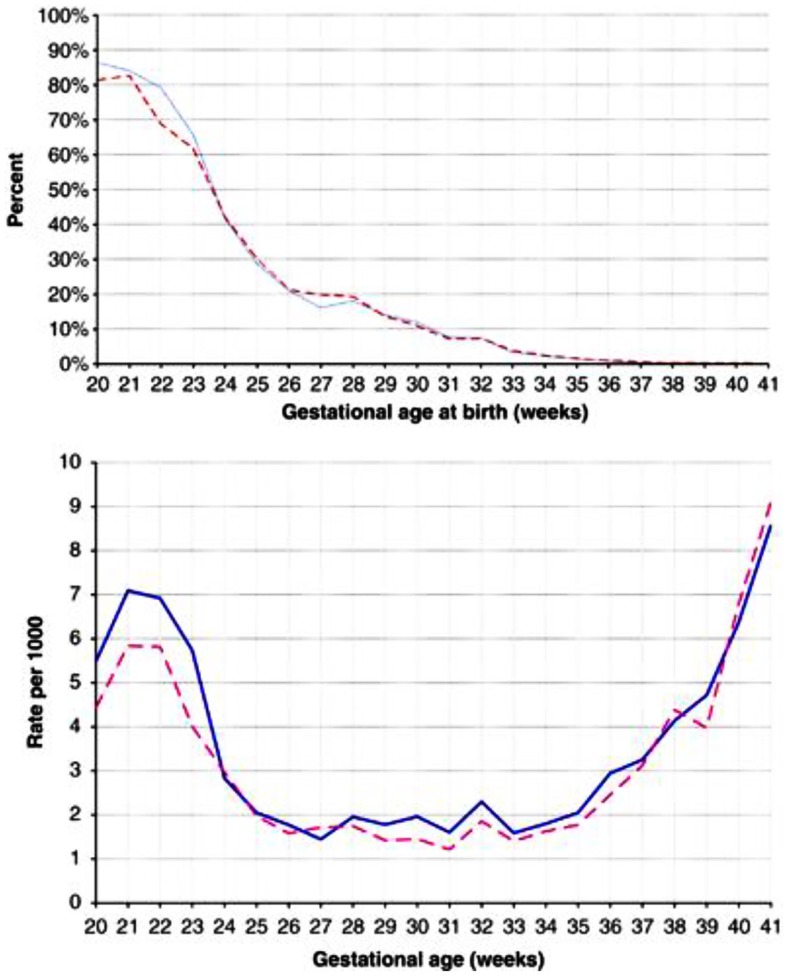
**(A)** Percentage of singleton stillbirths among all singleton livebirths and stillbirths (prevalence approach). Data are for males (solid blue line) and females (dashed red line), presented by gestational age. **(B)** Number of stillbirths per 1000 singleton livebirths and stillbirths combined (incidence approach). Data are for males (solid blue lines) and female (dashed red lines), presented by gestational age. Reproduced with permission (17).

For both SIDS and stillbirths, there is a male excess. In an early analysis of the sex ratio, the authors analyzed stillbirths in the United States from 1922 to 1936. The proportion of males at <16 weeks was nearly 80% but fell to 67% at 16 weeks, a low of 53.5% at 28 weeks but rose to 57% by 36–40 weeks (16) (Figure 2). In a recently reported analysis of birth outcomes in Canada between 2002 and 2007, 54.8% of males were stillborn compared to 51.4% of live births (17). The greatest difference between males and females appeared to between 20 and 24 weeks gestation as noted in the earlier study.

There is a rise in testosterone production associated with the period during which SIDS is most prevalent. Between 1 and 5 months, testosterone levels range from 0.03 to 6.14 nMol L^−1^ for males and 0.03 to 0.17 for females. In males, these levels decrease to 0.07–0.24 at 6–11 months. The ranges of testosterone in the adult females (<0.4 to 3.1 nMol L^−1^) tested in our studies were within the range for males in the 1- to 5-month age range. There was a positive correlation between testosterone levels and pro-inflammatory responses to LPS when the cells were pre-treated with IFN-γ or IFN-γ and a water soluble cigarette smoke extract (62).

**Figure 3:**
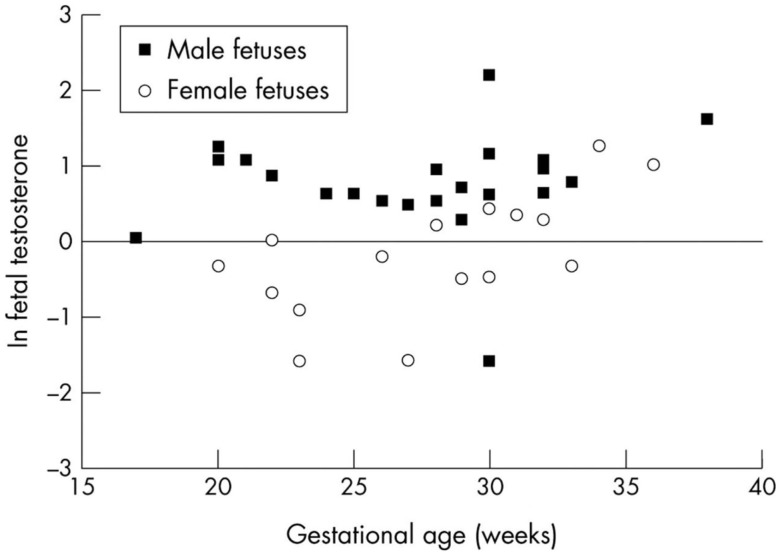
**Fetal testosterone levels, sex and gestational age**.

Fetal plasma testosterone levels for males were significantly higher (range 1.7–2.9 nMol L^−1^) than levels for females (range 0.45–1.3 nMol L^−1^) (85) (Figure 3). If testosterone has a similar effect on inflammatory responses in the fetus, male infants might have significantly higher pro-inflammatory responses to infection or bacterial components. We found many of the pro-inflammatory cytokines are higher in the amniotic fluid of males and the anti-inflammatory IL-1Ra significantly higher in females (Table 3).

**Table 3:** **Medians and ranges of cytokine levels (pg/ml) in amniotic fluid of male and female infants**.

Cytokine	Female (*n* = 12)	Range	Male (*n* = 12)	Range
IFN-γ	1373	15–6270	2717	96–14,417
IL1-β	28	<2.4–68	41	<2.4–121
IL-6	2270	292–10,738	1218	398–12,928
IL-8	1794	578–4185	2402	621–9179
TNF-α	351	311–2025	733	24–3176
IL-10	56	9–226	112	<1.8–303
IL1-Ra*	3908	1786–6295	1839	330–5064

* P < 0.001.

During the 20- to 24-week period when the difference between male and female stillbirths is most obvious, the difference in fetal testosterone levels is greatest. The testosterone levels rise significantly with gestational age among females but remain steady among males (Figure 3) (85). This raises the hypothesis that the higher testosterone levels present in males at 20–24 weeks gestation enhance pro-inflammatory responses as noted in our *in vitro* studies (62) and partly explain the excess of male stillbirths in this age range (Figure 2). Experimental systems are available to assess these interactions.

## Conclusion

There is evidence from a variety of sources to suggest infection and inflammation might play a role in fetal deaths. As a variety of micro-organisms has been identified in studies of stillbirths, the common thread is most likely the effects of the inflammatory responses to infection. There is evidence to support the hypothesis that risk factors associated with stillbirths could contribute to dysregulation of the balance of inflammatory responses to infection, and these responses might trigger physiological interactions leading to fetal loss. The following recommendations are derived from the assessment of how dysregulation of the inflammatory responses could help explain the risk factors associated with stillbirths.

1. Samples from both mother and infant need to be assessed by both conventional diagnostic methods and new molecular methods for evidence of infectious agents, particularly combinations of virus and bacteria.
2. Samples from both mother and infant need to be assessed for presence of bacterial toxins, both soluble and cellular, that can act as superantigens that can induce powerful cytokine responses.
3. Direct assessment of material from both mother and infant for evidence of pro-inflammatory and anti-inflammatory cytokines is needed.
4. Determination of cotinine levels in body fluids would help determine the level of exposure to cigarette smoke.
5. For both mother and infant, determine cytokine gene polymorphisms associated with high- or low-inflammatory responses and implicated in pre-term birth.
6. Experimental studies to assess further the interactions between genetic, developmental, and environmental risk factors for their role in dysregulation of inflammatory responses that could lead to infant death.

## Conflict of Interest Statement

The author declares that the research was conducted in the absence of any commercial or financial relationships that could be construed as a potential conflict of interest.
